# Barriers and facilitators to the integration of depression services in primary care in Vietnam: a mixed methods study

**DOI:** 10.1186/s12913-018-3416-z

**Published:** 2018-08-16

**Authors:** Jill Murphy, Kitty K. Corbett, Dang Thuy Linh, Pham Thi Oanh, Vu Cong Nguyen

**Affiliations:** 1 Centre for Applied Research in Mental Health and Addiction, Faculty of Health Sciences, Simon Fraser University, Suite 2400, 515 W. Hastings Street, Vancouver, BC V6B 5K3 Canada; 2 School of Public Health and Health Systems, University of Waterloo, 200 University Ave. West, Waterloo, ON N2L3G1 Canada; 3 Institute of Population, Health and Development, 18 Lane 132, Hoa Bang, Yen Hoa, Hanoi, 122667 Vietnam

**Keywords:** Depression, Primary care, Global mental health, Implementation, Vietnam, Mixed methods

## Abstract

**Background:**

Although the prevalence of depression in Vietnam is on par with global rates, services for depression are limited. The government of Vietnam has prioritized enhancing depression care through primary healthcare (PHC) and efforts are currently underway to test and scale-up psychosocial interventions throughout the country. With these initiatives in progress, it is important to understand implementation factors that might influence the successful integration of depression services into PHC. As the implementers of these new interventions, primary care providers (PHPs) are well placed to provide important insight into implementation factors affecting the integration of depression services into PHC. This mixed-methods study examines factors at the individual, organizational and structural levels that may act as barriers and facilitators to the integration of depression services into PHC in Vietnam from the perspective of PHPs.

**Methods:**

Data collection took place in Hanoi, Vietnam in 2014. We conducted semi-structured interviews with PHPs (*n* = 30) at commune health centres and outpatient clinics in one rural and one urban district of Hanoi. Theoretical thematic analysis was used to analyse interview data. We administered an online survey to PHPs at *n* = 150 randomly selected communes across Hanoi. *N* = 226 PHPs responded to the survey. We used descriptive statistics to describe the study variables acting as barriers and facilitators and used a chi-square test of independence to indicate statistically significant (*p* < .05) associations between study variables and the profession, location and gender of PHPs.

**Results:**

Individual-level barriers include low level of knowledge and familiarity with depression among PHPs. Organizational barriers include low resource availability in PHC and low managerial discretion. Barriers at the structural level include limited mental health training among all PHPs and the existing programmatic structure of PHC in Vietnam, which sets mental health apart from general services. Facilitators at the individual level include positive attitudes among PHPs towards people with depression and interest in undergoing enhanced training in depression service delivery.

**Conclusions:**

While facilitating factors at the individual level are encouraging, considerable barriers at the structural level must be addressed to ensure the successful integration of depression services into PHC in Vietnam.

**Electronic supplementary material:**

The online version of this article (10.1186/s12913-018-3416-z) contains supplementary material, which is available to authorized users.

## Background

Although depression is a major contributor to the disease and disability burden in low and middle-income countries (LMICs) [1, 2], access to evidence-based care is critically low. In response to this gap, integrating mental health services into primary healthcare (PHC) through approaches such as task-sharing have been recommended [3]. During the last decade, increased research investment in global mental health has led to an enhanced evidence base about the effectiveness of such approaches for treating depression in the community [4]. Little is known, however, about how to implement and scale-up these interventions into “real-world” settings. Enhancing the role of implementation research in global mental health has been identified as crucial [5]. Understanding barriers and facilitators to the integration of mental health services in PHC can inform the implementation and scale-up of these interventions, enhancing treatment availability and sustained delivery of care for those suffering from depression.

Vietnam is a lower-middle income country [6] with a population of approximately 91 million people [7]. While mental health epidemiological evidence is limited for Vietnam, studies suggest that prevalence of depression is considerable [8], with rates of over 20% identified in some populations [9–11]. Mental health services are predominantly limited to tertiary care services for psychotic disorders and epilepsy [12, 13]. The Vietnamese government introduced the National Community Mental Health Program (CMHP) in 2000 [14] to enhance the screening and treatment of people with mental illness in PHC. In practice, the activities of the CMHP are limited to referrals and provision of medications, with one member of staff at each health centre, usually a physician’s assistant, given responsibility for the program. Ng et al. [14] found that although the CMHP operates throughout Vietnam, it covers only 64% of the national population, with 63% of enrolled patients treated for schizophrenia, 35% for epilepsy, and only 2% for depression. The exclusion of high prevalence conditions is a considerable shortcoming and points to limited capacity to manage symptoms of depression in PHC [13, 15]. The government of Vietnam has also developed a National Mental Health Strategy for 2015–2020, through which it continues to work towards the enhancement of community-based services for mental illness [8].

As the government of Vietnam takes steps towards improving depression services, it is important to identify factors that might impede or facilitate the integration of services for depression in PHC. Primary healthcare providers (PHPs) are the frontline of PHC delivery, providing care to patients with general health concerns, managing health promotion and prevention programs, and providing antenatal care. As services for patients with symptoms of depression are introduced, PHPs will also be responsible for the delivery of these services. The perspective of PHPs is therefore essential in the process of planning for depression service integration in PHC. We conducted this study in tandem with a pilot study and subsequent randomized controlled trial (RCT) to test the effectiveness of a Supported Self-Management intervention for depression, delivered in PHC and community-based setting in Vietnam [16, 17]. This initiative is taking place in partnership with Vietnam’s Ministry of Labour, Invalids and Social Affairs (MOLISA) in alignment with their objective to improve community-based care for depression, with a view to scaling-up the intervention throughout the country.

This study examines barriers and facilitators to the integration of services for depression in PHC from the perspective of PHPs in one rural and one urban district of Hanoi municipality, Vietnam by examining individual, organizational and structural variables. Understanding barriers and facilitators to the delivery of these services from the perspective of PHPs can help to identify and mitigate challenges to implementation, leverage existing opportunities for success, and inform planning for further scale-up and sustainability.

### Theoretical approach and variables of interest

Barriers and drivers to the integration of mental health services into PHC may exist at several levels of a health system. In this study we examine the perspectives of PHPs, whose individual experience, characteristics and circumstances might facilitate or impede the successful integration of depression services. PHPs are also influenced by factors at the organizational level and in the structural environment that might impact their ability to integrate depression services into their day-to-day routines [18–20]. Contextual Interaction Theory (CIT) [21] is helpful for understanding implementation factors at the individual, organizational and structural levels. The theory “assumes that the course and outcome of the policy process depend not only on inputs…,but more crucially on the characteristics of the actors involved, particularly their motivation, information and power” ([21], 290). The theory was designed to examine how the motivation, information and power of actors, and the nature of their interaction with other actors, might predict the outcomes of the policy process, and is also suitable for assessing implementation outcomes. *Motivation* can be defined as internal (e.g., attitudes, self-efficacy) and external (e.g. normative, economic, social, political variables). *Information* includes technical knowledge, general knowledge about the policy or program being implemented, about how to comply with the policy or program, and access to information. *Power* can be understood in terms of capacity (e.g. financial, personnel, time) or control. Control may be formal, based on legislation, regulation or formal roles in an organization, or may be informal, related to perceptions of leadership, influence, etc. [21–23]. While this study is concerned with identifying barriers and facilitators from the perspective of only one group (PHPs), CIT nonetheless proved useful for informing the design of the study and framing the analysis, enabling us to identify barriers and facilitators at individual, organizational and structural levels and to understand the broad context in which these barriers and facilitators take place. This in turn allowed us to elicit a comprehensive understanding of the ways in which system level factors interact with the day-to-day experience of PHPs including the organizational context of CHSs, and their individual knowledge and attitudes about mental illness and depression. CIT is therefore a useful framework for simultaneously focusing on one group of actors and capturing the influence of the broader social and health system context.

As part of the exploratory phase for this study, we conducted a narrative review of the literature identifying barriers and drivers to the integration of mental health services in primary care in both LMIC and high-income country settings. This review led to the identification of a number of variables that might act as barriers and drivers to integrating mental health services in PHC. While a comprehensive description of the results of this review is beyond the scope of this paper, these variables are displayed in Table 1.

**Table 1:** Hypothesized study variables and analytical framework

	Level
Motivation	
Internal	
Attitude, Stigma and Discrimination	
Familiarity with people with mental illness (depressive symptoms) [40, 42]	Individual
Explanatory models and health beliefs (including aetiological beliefs)^a^	Individual
Perceived need/ perception of mental illness as significant in primary care [38]	Individual
Perceived role and workload [38, 43–45]	Individual
Perceptions of people with mental disorders (including characterizations of people with mental illness and social distance measures) [38, 40, 43, 46]	Individual
External	
Social/ cultural environment and norms^a^	Structural
National or organizational priorities in the health sector [22, 39, 47]	Structural
Information	
Training in mental health [12, 38, 42, 43, 47–49]	Individual/Structural
In-service training and ongoing supervision [50, 51]	Individual/Organizational/Structural
Power	
Capacity	
Resource availability: medicines and equipment, financial, personnel, space, etc. [39, 47, 52]	Organizational
Perceived workload [39, 53]	Individual/ Organizational
Perceived self-efficacy [38, 43, 44, 53]	Individual
Control	
Leadership or champion within organization [52]	Organizational

^a^Results related to this variable are published elsewhere (Murphy et al., [16])

## Methods

Data collection for this study took place from August to December 2014 in Hanoi, Vietnam and consisted of semi-structured interviews and an online survey. Mixed methods can provide a deeper understanding of phenomena and allow for triangulation of theoretical, qualitative and quantitative results [24]. In this study we used a predominantly parallel mixed methods approach [24, 25], with data collection and analysis taking place separately and compared and consolidated during the interpretive phase. During data interpretation, we used the qualitative results to deepen our understanding of the quantitative survey results. We used CIT and the individual, organizational and structural-level variables that emerged from the literature review (Table 1) to design the interview guide and survey, described further below.

### Interviews

The first author conducted thirty semi-structured interviews with the support of co-authors DTL and PTO, who are bilingual in Vietnamese and English. We conducted interviews at eight Commune Health Stations (CHSs) and two Outpatient Clinics (OPCs) in one urban (Dong Da) and one rural (Thach That) district of Hanoi, which has a population of approximately 7 million inhabitants [26]. The majority of PHPs interviewed (*n* = 27) were female, which is representative of the composition of staff at the centres included in this study. Interview respondents were: doctors (*n* = 8), nurses (*n* = 11), physician’s assistants (PAs) (*n* = 8) and pharmacists (*n* = 3). Seventeen respondents were based in the rural area, while 13 were in the urban district. Due to the strict government permissions required to conduct interviews at CHCs and OPCs and the unpredictability of the PHPs’ schedules, purposive sampling was used and respondents participated in the study based on availability. Interviews were conducted in private rooms at the health centres, with the primary researcher and research assistant present.

The interview guide (see Additional file 1) and consent forms were translated from English to Vietnamese and then back translated for semantic equivalence. We conducted two test interviews with one doctor and one nurse, and made adjustments as necessary to the interview questions. The term “Common Mental Disorders” did not seem to be understood by respondents and risked confusing them. We therefore replaced the term with “depression” *(trầm cảm),* which is a specific term that is understood by PHPs. The interview guide can be found in Additional file 1. Twenty-four interviews were recorded. Six respondents preferred not to have their interview recorded and in these cases extensive field notes were taken. The recorded interviews were transcribed in Vietnamese by two research assistants and subsequently translated from Vietnamese into English for analysis.

We analysed interview data using a theoretical thematic analysis approach [27], allowing for analysis to be structured deductively around the initial variables of interest, with additional themes identified as they emerged throughout the analysis process. Analysis began with immersion in the data through thorough reading and rereading of the transcripts. We developed a codebook using the initial variables of interest, and added additional codes as they emerged. We coded the data using NVivo 10 software [28]. Themes were identified from within each category of the codebook, and were then reviewed, refined and analysed in detail [27]. The results of analysis of the 30 interviews in terms of coherence and coverage of emerging patterns suggest that thematic saturation was achieved [29].

### Survey

We implemented a survey with PHPs in Hanoi, including primary care physicians, PAs and nurses. Inclusion criteria for the survey included being employed as a physician or physician’s assistant in a CHS at the time of the survey. We used a list of all commune health centres in Hanoi (*n* = 579) as the sampling frame,^1^ selecting every fourth centre for a total of *n* = 150 communes or 26% of communes in Hanoi. A research assistant (PTO) then contacted each CHS in the sample by telephone, requesting that they participate in the survey. On the recommendation of the Vietnamese research team, we included a small incentive for participation (50,000 VND in mobile phone credit, equivalent to USD $2.50), which is common practice in the Vietnamese context.

The sample size for survey participants was calculated using an 80% confidence interval with a margin of error of + − 5. The targeted sample sizes for each population were determined to be: physicians (*n* = 130), and nurses and PAs (*n* = 150). The 80% C.I. was chosen in order to balance accuracy with feasibility, given both time and resource constraints (Cocks and Torgerson, 2013). The response rate was higher than anticipated, with 331 responses received. Table 2 displays the type, gender distribution and location (urban or rural) of providers that responded to the survey. We received responses from 17 rural districts and 12 urban districts of Hanoi.

**Table 2:** Professional characteristics of the survey sample by gender

	Female (*N* = 147)	Male (*N* = 80)	All^a^
Profession			
Physician	47 (66.2%)	24 (33.8%)	71 (31.4%)
PAs	63 (57.3%)	47 (42.7%)	110 (48.6%)
Other^b^	32 (78.0%)	9 (21.9%)	41 (18.1%)
Total^a^	142	80	222
Urban or rural			
Urban	38 (73.0%)	14 (26.9%)	52 (23.0%)
Rural	108 (62.0%)	66 (37.9%)	174 (76.9%)
Total^a^	146	80	226

^a^Total varies due to missing data

^b^Nurses (66%), midwives (10%), TVM practitioners (5%), unspecified (19%)

The survey instrument (Additional file 2) included yes/no answers, Likert scales and open-ended fill-in questions. We developed a draft survey prior to conducting field research and slightly revised it based on emerging data from the qualitative interviews to better define mental illness and common mental disorders including depression. We also added questions based on additional contextual knowledge gained from the surveys, such as the role that commune centres play in patient referral, distribution of medications, and offering health promotion and prevention programs. We designed the survey to elicit responses related to the variables presented in Table 1.

We distributed the survey using email and SMS text message, which is widely utilized by health workers in Vietnam. The survey included a consent page followed by a declaration of anonymity and confidentiality. Survey responses were monitored for volume of completion with reminders sent 2 weeks following the initial distribution. As described above, the number of respondents exceeded expectations.

We used descriptive statistics to describe the extent to which the variables of interest may act as barriers and facilitators to integrating services for depression in primary care. A chi-square test of independence indicated where there was a statistically significant (*p* < .05) association between the variables of interest and the profession, location and gender of PHPs.

### Ethical approval

Ethical approval was obtained by the research ethics boards of Simon Fraser University in Canada and the Institute of Population, Health and Development in Vietnam.

## Results

### Familiarity with depression

The survey results show that PHPs had low levels of familiarity with depression among their patients; see Table 2. Of surveyed PHPs, 64% indicated that depression was not widespread among patients visiting CHCs. When asked about depression in the general community, responses suggest that they perceived a higher presence of depression in the community compared with the patient population (Table 3). As shown in Tables 2 and 3, in reference to both patient and community populations, physicians estimated the prevalence of depression to be higher than other PHPs, with a statistically significant difference based on profession [Patient prevalence: X^2^(2, *N* = 222) = 17.314, *p* < .008; Community prevalence: X^2^(2, *N* = 223) = 28.3482, *p* < .000].

**Table 3:** Perceived depression prevalence in CHS patient population

In your opinion, how widespread (WS) are CMDs like depression among the patients that visit your CHS?						
	Very Widespread (%)	Moderately Widespread (%)	Not Widespread (%)	N/A	Row N	Chi-square and *p*-value
Profession						
Physicians	16%	25%	58%	1%	71	
PA’s	3%	20%	71%	6%	110	
Other	2%	24%	63%	10%	41	
All	7%	23%	64%	5.7%	222	17.3145; .008^**^
Location						
Urban	2%	14%	78%	6%	50	
Rural	8%	25%	63%	5%	177	
All	5%	19.5%	70.5%	5.5%	227	5.4683; .141^*^
Gender						
Female	8%	18%	69%	5%	147	
Male	4%	31%	60%	5%	77	
All	6%	24.5%	64.5%	5%	224	6.1118; .106^*^

^*^The result is not significant at *p* < .05

^**^The result is significant at *p* < .05

The belief that depression is higher in the community than in the patient population is consistent with interview results. Interviews further suggest that PHPs believe there is a gap in help seeking for depression among community members:

Well, there are patients coming to the CHS to get the medicine …There are also other people in the community who have symptoms of mental disorder but they don't want to disclose about that, they hide it. They don’t go to the CHS but when we look at them we know they have mental disorders. (I-08, Nurse, rural area)

### Training

Survey results indicate that physicians have received more pre- and in-service mental health training than other PHPs, with a statistically significant difference by profession [X^2^ (2, *N* = 224) = 14.0415, *p* < .001] for pre-service training (Table 4). A statistically significant difference by gender [X^2^ (2, *N* = 225) = 28.6522, *p* < .00001] for in-service training was also found. All PHPs surveyed indicated that they would like to learn more about diagnosing and treating people with mental illness, with 75% selecting “strongly agree” (Table 4).

**Table 4:** Perceived prevalence of depression in community population

In your opinion, how prevalent are CMDs like depression in the community in general?							
	High prev.	Mod. high prev.	Mod. low prev.	Very low prev.	N/A	Row N	Chi-square and *p*-value
	(%)	(%)	(%)	(%)	(%)		
Profession							
Physicians	19%	33%	33%	15%	0%	69	
PAs	3%	37%	35%	24%	2%	113	
Other	0%	29%	51%	15%	5%	41	
All	11%	33%	39%	18%	3.5%	223	28.3482; .000^**^
Location							
Urban	2%	37%	40%	21%	0%	52	
Rural	9%	33%	36%	19%	2%	177	
All	5.5%	35%	38%	20%	1%	229	4.343; .362^*^
Gender							
Female	8%	32%	36%	21%	3%	145	
Male	6%	36%	39%	19%	0%	80	
All	7%	34%	37.5%	20%	1.5%	225	3.0946; .542^*^

^*^The result is not significant at *p* < .05

^**^The result is significant at *p* < .05

Interview participants described the training they receive in mental health. Physicians said they received approximately 4 weeks of required theory and clinical training, while nurses received minimal training that was limited to theory with no clinical practice. Pharmacists received general training and did not spend dedicated time learning about psychopharmacology. Many PAs described receiving no mental health training during vocational college. In-service mental health training was only provided to physicians who also manage CHSs and the PA who is responsible for the CMHP.

Responses of PAs in interviews and in surveys differed. Although 77% of PA’s indicated in the survey that they had received pre-service mental health training, in interviews many PA’s stated that they did not receive specific mental health training in vocational college:

When I was a student… there were some lessons on neurology but not about mental problems… It's only when I started this job in the [CMHP] that I learned more about mental health. (I-04,PA, rural area)

The PHP responsible for the CMHP was not required to have any previous or concentrated training in mental health, but rather received training only when they became responsible for the program:

When I started working, I used to work in that [CMHP] program but my main responsibility was to receive and provide medication for the patients so I don't really have deep knowledge about mental illness. (I-28, PA, urban area)

Similarly to survey participants, despite low levels of knowledge and familiarity with people with mental illness, interviewed PHPs expressed interest in receiving more training in this area. PHPs demonstrated enthusiasm for more training opportunities and described the need for better training for health workers:

Here we always want to improve people's awareness about mental illness, so that they can know about the cause of the problems, and we can have a better treating method that addresses the right causes. (I-15, Physician, rural area)*.*

### Perceived role and workload

When asked if they felt they had the ability to learn new skills given their workload 95% of surveyed PHPs either strongly or somewhat agreed, with no statistically significant difference between profession, gender, or location (no table shown^2^). In open ended questions, surveyed participants described advantages of patients receiving services at the PHC level, noting that PHC centres are optimal places for patients to access care for depression, because they are more accessible both geographically and financially for patients who are poor:

Patients with mental disorders are very poor, so taking them to the commune health center will help them get better care. (S-93, Survey participant)

Survey participants also used open-ended questions to comment on their workload:

Too many health programs at the same time are constraining our capacity to take care of people's health. Unnecessary programs should be cut off. (S-57, Survey participant)

It is necessary to reduce paperwork so that we can focus more on taking care of the people’s health. We are now under too many health programs, which cause a lot of stress on daily work. (S-24, Survey participant)

Despite being willing and able to take on additional training and skills, interviewed PHPs described the role of diagnosis and treatment of mental disorders as belonging to specialists, while their role is to provide referrals for more specialized services and to offer complementary care including advice and supplementary medications (e.g. sleeping pills, vitamins and traditional remedies).

For patients with a mild mental health problem, we introduce about the problem and counsel them to take some support medicine, like medicine for the neurological system, to improve the brain blood circulation so that the patient can get rid of the headache^3^. If we know exactly the patient’s problem, we will advise them to go an appropriate level of health care. (I-20, Physician, urban area)

Some PHPs also associated care for patients with mental health problems only with the CMHP, and referred to work with patients with mental health problems as “not my job”. PHPs stated that making referrals, even with mild cases of mental illness, is required under the CMHP:

Well, even for mild mental disorders, we still need to refer the patient to a higher level so that the doctor there can diagnose if the patient is really having a mental problem. (I-26, Nurse, urban area)

PHPs also displayed a willingness to take on the role of care providers to people with depression. Similarly to survey participants, when asked about their workload, interview participants expressed their willingness to learn and take on new tasks despite being responsible for patient care and numerous programs.

### Attitudes

We included questions related to social distance and attitudes toward people with depression in the survey to elicit anonymous responses that might not emerge during interviews due to social desirability bias. As shown in Table 5, most respondents showed a high level of acceptance for people who had experienced depression: 77% strongly agreed that they would be willing to spend time socially with a friend who was diagnosed with depression. 39.5% strongly agreed and 48.5% somewhat agreed that they would be confident in the ability of a colleague with depression to do her job effectively.

**Table 5:** PHP reported pre- and in-service training in mental health and interest in additional training

	Received pre-service training about mental health				Received in-service training about mental health				I would like to learn more about diagnosing and treating people with depression				
Profession	Yes (%)	No (%)	Row N	Chi-square and *p*-value	Yes (%)	No (%)	Row N	Chi-square and *p*-value	Strongly agree (%)	Somewhat Agree (%)	Disagree^+^ %	Row N	Chi-square and p-value
Physicians	95	6	71		83	17	71		78	19	3	68	
PA’s	77	23	113		67	33	113		71	29	1	112	
Other	68	33	40		72	28	39		78	22	0	41	
All	80	2	224	14.0425; .001^**^	74	26	223	5.6048; .061^*^	75	24	1	221	4.8587;.562^*^
Location													
Urban	77	23	52		75	25	52		69	28	3	51	
Rural	81	19	177		71	29	176		76	23	1	175	
All	79	21	229	0.3746; .541^*^	73	27	228	0.3145; .575^*^	72.50	25.50	2	226	4.268; .234^*^
Gender													
Female	82	18	146		73	27	145		72	26	1	145	
Male	80	20	80		71	29	80		81	18	1	78	
All	81	19	226	0.1641; .685^*^	72	28	225	28.6522; <.00001^**^	76.50	22	1	223	2.7656; .429^*^

^*^The result is not significant at *p* < .05

^**^The result is significant at *p* < .05

^**+**^Somewhat disagree and strongly disagree categories were collapsed as cell size was too small for *p*-value calculation

The attitudes expressed by interviewees toward patients with mental illnesses reveal perceived challenges PHPs have working with these patients. They described the need to approach patients gently, tactfully and, at times, cautiously. Several interviewees stated that this approach was necessary due to the perceived volatility of such patients and the challenges of “coordinating” with them.

PHPs also described a number of skills and characteristics they believed were necessary for interacting with patients with mental illness. They described the skills needed to work with patients with mental illness as effective communication, patience, tact, and understanding:

Well…most of them [patients with depression] feel very tired, they have lost their passion. I don't think there is anything negative. But they don't want to communicate with people, they are afraid to. We need to be tactful to get information from them. Otherwise they will not coordinate with us. That's a challenge for people working with mental illness patients. We need to have a kind manner when working with them. (I-20, Doctor, urban area)

### Resources

PHPs described several resource needs regarding mental health services in primary care. Although 81% of survey respondents reported that private space was necessary for consulting patients with depression, many PHPs indicated that private space was unavailable at their health centre (Table 6). Although physicians indicated a higher availability of private space than other PHPs (66% vs. 51% for PAs and 47% for other providers), the results were not statistically significant. There was a statistically significant difference by location: X^2^(2, *N* = 213) = 11.7703, *p* < .05. While rural respondents’ yes responses were somewhat higher (56%) compared with urban respondents (51%), 22% of urban respondents indicated that private space was sometimes available, compared with 6% of rural respondents.

**Table 6:** Social distance measures

	I would be willing to spend time socially with a friend who has been diagnosed with depression					I would have confidence in the ability of a colleague who had had depression to do her job effectively				
	Strongly agree (%)	Somewhat agree (%)	Disagree^+^ (%)	Row N	Chi-square and *p*-value	Strongly agree % (N)	Somewhat agree % (N)	Disagree^+^ % (N)	Row N	Chi-square and *p*-value
Profession										
Physicians	76	23	0	70		44	44	11% (8)	70	
PA’s	72	24	4	111		35	52	12% (14)	113	
Other	85	15	0	41		46	39	15% (6)	41	
All	77	21	2	222	4.2537; .642^*^	42	45	12.50	224	4.2537; .642^*^
Location										
Urban	79	19	2	52		39% (20)	50% (26)	10% (6)	52	
Rural	74	23	3	175		40% (70)	47% (83)	13% (23)	176	
All	76.50	21	2	227	0.9699; .809^*^	39.50%	48.50%	13%	228	0.1607; .984^*^
Gender										
Female	78	19	3	145		40% (58)	46% (67)	14% (20)	145	
Male	73	25	0	79		41% (33)	48% (38)	11% (9)	80	
All	75.50	22	1.50	224	4.7084; .194^*^	40.5	47%	13%	225	0.509; .917^*^

^*^The result is not significant at *p* < .05

^+^Somewhat disagree and strongly disagree categories were collapsed as cell size was too small for *p*-value calculation

The need for more staff was also described, especially in relation to the demands of numerous programs. As shown in Table 6, survey participants overall reported that they would be able to provide better care to patients with depression if there were more staff. There was a statistically significant difference by profession, with physicians indicating more strongly than PAs and other PHPs (78%% vs. 50 and 39%) that more staff are needed [X^2^(2, *N* = 222) = 43.7232, *p* < .00001].

As one interviewee stated, the fact that there was only one physician in each PHC centre and PAs were minimally trained was a challenge for mental health care delivery:

I am the only doctor here, while there are so many health programs, 36 programs with many tasks and patients to check. Therefore, PAs are in charge of checking the patients as well. However, their capacity is limited, leading to many difficulties in checking and treating patients with mental illness. (I-15, Doctor, rural area)

## Discussion

This study explored barriers and facilitators to the integration of services for depression in primary care in Vietnam. We found that several barriers exist, particularly at the organizational and structural levels. Using the construct of CIT, the *information* of PHPs is limited, and the *power-control* of organizations is also low. At the structural level, *external motivation* is low. Despite these barriers, PHPs displayed a high level of *internal motivation,* identifying the need for improved depression service delivery at the PHC level, and showing an interest in enhanced training and capacity building to enable them to take on this role.

### Barriers

Knowledge of and familiarity with depression are low among PHPs. This is consistent with research among community based health workers elsewhere in Vietnam [30]. Compared with epidemiological data, PHPs underestimated the prevalence of depression in the community and likely underestimated prevalence in their patient populations. Biomedical help-seeking for all but the most severe psychiatric symptoms has been found to be low in studies in central [31] and northern [30] Vietnam, which might contribute to both the underestimation of and lack of familiarity with depression among PHPs.

While our questions in both the survey and interviews focused on depression, during interviews PHPs frequently referred to mental illness in general. This likely also reflects a lack of knowledge of depression. Though it might suggest that they tend not to distinguish among discrete conditions when discussing mental health, in a related study [32] we found that although familiarity with depression is low, PHPs in Vietnam do demonstrate an understanding of depression as a discrete condition. Neimi et al. identified low levels of knowledge of depression among health workers in northern Vietnam [30]. Low levels of knowledge about mental illness among PHPs has also been identified in other contexts, in both high-income [33] and low-income settings [34, 35].

While the majority of PHPs indicated that they received some mental health education during their pre-service training, it was often minimal. This lack of training affects mental health knowledge and perceived self-efficacy among PHPs. Given the structure of the mental health system and its emphasis on treating schizophrenia and epilepsy, it is unlikely that even physicians receive adequate training about depression or about psychosocial approaches to treatment. Other PHPs receive minimal mental health training, with PAs relying on in-service mental health training only if they are allocated responsibility for the CMHP. Low levels of training, including both pre- and in-service training, have been identified as a barrier to the integration of mental health services into PHC in numerous studies from LMICs, including Cambodia [34], Ethiopia, India, Nepal, Uganda and South Africa [36], Zambia [35] and Mexico [37]. The need for improved mental health training for PHPs is also not limited to low- and middle- income settings, and has been identified in studies from high income setting including Canada [33].

PHPs in the study saw detection and treatment of CMD as the role of specialists, while their role was to make referrals or provide complementary treatments. This is consistent with the findings of a study about PHP attitudes to mental health services provision in Kenya [38]. However although some PHPs described caring for patients with mental illness as not being part of their role, this might be a reflection of the structure of the CMHP, which specifically designates one staff member to the program, rather than an explicit reluctance to take on this work. PHPs indicated that PHC centres are more accessible to patients due to the location within communities and were most accessible to patients with low socioeconomic status. They indicated that with enhanced training and support, services for depression could effectively be delivered in PHC. In a study of PHPs attitudes toward integration of mental health services in Cambodia, Alfredsson et al. [34] also found that PHPs believe that providing mental health care in PHC would be valuable due to the accessibility of PHC centres.

Resources and competing workloads were identified as barriers to integration at the organizational level. Private space for consultation with patients with CMD and numbers and capacity of personnel were described as resource gaps, which is consistent with studies from other regions [37]. A statistically significantly higher proportion of physicians indicated that more staff is necessary for providing better care. As managers, physicians would be most aware of these staffing challenges, as each CHC has only one physician, leaving much of patient care to PAs who might receive no training in mental health. Each PHP is also responsible for several programs, in addition to routine patient care, emergency care, and administrative and maintenance tasks. High workloads and competing priorities were also identified as a barrier in a Kenyan study of the perspectives of PHPs about integrating mental health services in primary care [39].

At the structural level, a number of barriers related to the structure of PHC in Vietnam emerged from the study. PHPs described being overwhelmed by the number of programs offered and the demand this places on their workload. Some indicated that the number of programs took away from their ability to provide patient care. The inability of physician managers to determine program or policy direction may also be a barrier. While physicians supported the need for enhanced mental health services, they were constrained by the top-down nature of primary care delivery in which programs are the mandate of the higher levels of the health system.

The CMHP represents an important step towards providing care for mental illness in primary care, but it has several limitations that appear to impede the satisfactory integration of mental health services in primary care. Because PHPs seem to associate “mental health” only with the CMHP, they do not see mental health care as within the purview of general health care services. The majority of patients enrolled in the CMHP are diagnosed with schizophrenia or epilepsy, while depression is rarely treated under the program despite having much higher prevalence [14]. This means that depression and other CMDs likely go undetected and untreated. Despite the CMHP’s goals, in practice treatment is almost exclusively limited to referring patients to tertiary care and the subsequent distribution of medications. The in-service mental health training delivered under the CMHP is provided only to health centre managers and the staff member responsible for the program, excluding other PHPs. This limits the mental health knowledge and training of staff and acts as a barrier to integration of mental health services in PHC.

### Facilitators

Regardless of having low knowledge about and familiarity with patients with mental illness, PHPs did not display overtly negative attitudes toward patients with depression. Social distance scores suggest that PHPs would be comfortable to socially interact and work with people who have experienced depression. PHPs did describe challenges related to working with patients enrolled in the CMHP, many of whom live with schizophrenia. Some PHPs described these patients as volatile and uncooperative, pointing to the need for enhanced training, support and supervision. Similar attitudes were identified in a Cambodian study [34]. PHPs also described patients with mental illness as vulnerable and requiring a tactful, gentle approach to care. While perceptions of people with mental illness as weak have been associated with increased stigma [40] this attitude may also suggest empathy towards these patients.

Despite heavy workloads, numerous responsibilities, and other barriers, PHPs displayed an interest in and willingness to learn more and provide care for patients with depression. They recognized the need for more training in mental health and welcomed new opportunities to obtain this training. This positive attitude towards depression care provision among PHPs despite numerous barriers was found in other studies from Cambodia [34], Nepal [41] and Zambia [35]. This presents an important opportunity for the further integration of mental health services in primary care; the PHPs in this study were willing and interested to enhance their capacity and thought that, given the right skills and supervision, they could be effective and appropriate providers of care for depression. This positive attitude and openness among PHPs is an important facilitator.

### Limitations

This study took place in Hanoi, and it might not be generalizable to the rest of the country. The analysis did, however, lead to theoretical saturation and we believe that the results are representative of the experience of PHPs working in the Hanoi district.

Our study focused on perspectives of PHPs and did not examine perspectives of patients and policy-makers. Further research that includes other stakeholders would lead to a more comprehensive picture of barriers and facilitators to integrating depression services in PHC. Despite this limitation, because PHPs act as brokers between communities and the healthcare system and are the front line of PHC delivery, their perspectives are instrumental to understanding and improving depression service integration.

An additional limitation is the chance of errors and misunderstandings associated with the cultural origin and linguistic limitations of the primary author. This risk has been mitigated by the iterative nature of the research process and the collaboration of Vietnamese colleagues, who were engaged throughout in developing study materials, collecting data, contextualizing participants’ input, and co-authoring this paper.

Finally, the use of purposive sampling may have acted as a limitation in this study. Due to the context of Vietnam, where we were required to use formal channels to access PHPs, this approach was necessary. We are confident however that this approach did not limit the validity of our findings.

### Study implications

The findings of this study are consistent with studies from Vietnam and from other LMICs. This study, however, is unique in that it assesses barriers and facilitators to the integration of depression services into PHC at the individual, organizational and structural levels. It shows that despite a considerable facilitator at the level of individual providers, structural-level barriers must be addressed in order to achieve successful integration of depression services into PHC.

The integration of depression services into PHC is an evidence-based practice that is recommended to address the mental health treatment gap [3]. CHSs are embedded within their communities and, as PHPs describe, are convenient and accessible for patients. The enhancement of the capacity of CHSs to provide patient care, including mental health care, would be beneficial to patients and welcome to PHPs. The main facilitator to the integration of depression services in primary care identified in this study is the willingness and interest of PHPs to learn more about depression and to take on a greater role in the provision of depression services. Enhanced training, including pre-service and in-service training for all PHPs, is essential to achieving this. This must include training about depression and about psychosocial approaches to care. In addition, the CMHP must be expanded to include depression and other common mental disorders, including enrolment of patients with depression and other CMDs in the program, the provision of antidepressant medications in PHC, and ongoing training and supervision for PHPs. These steps are essential to further promote, rather than impede, the true integration of mental health care into PHC.

## Conclusions

It is promising that the government of Vietnam continues to prioritize enhanced community-based care for mental illness, including depression, and that they are supportive of adopting psychosocial interventions that can take place at the level of PHC. This commitment, however, does not appear to have been realized yet. The program and policy directions of PHC centres in Vietnam are determined in a top-down manner, meaning that the discretion of PHPs to implement change is limited without clear policy direction from higher levels within the system. For depression services to be fully integrated into PHC, it is necessary that mental health, especially detection and treatment for common mental disorders, be reframed as part of the general health services offered by all PHPs. Given low levels of help seeking, community outreach and awareness initiatives should be introduced as part of enhanced mental health services in primary care. With a commitment to training PHPs in screening and the implementation of low-cost psychosocial interventions, and investment in community outreach and awareness, services for depression promise to be better integrated into primary care in Vietnam.

While this study is specific to Vietnam, it has implications for the broader field of global mental health. Questions of implementation, including how to promote effective delivery and scale-up of mental health interventions in LMICs, are essential in this field [5]. Both the approach used and the findings of this study contribute to the evidence base of global mental health implementation, demonstrating the importance of a multi-level, mixed methods analysis for understanding barriers and facilitators to mental health service implementation.

## Additional files

Additional file 1 Qualitative interview guide, this file contains the English language version of the semi-structured interview guide used to conduct the qualitative component of this study in Hanoi, Vietnam. (DOCX 17 kb)

Additional file 2 Study survey instrument. (PDF 801 kb)

## Abbreviations

CHS: Commune Health Station
CI: Confidence intervals
CIT: Contextual interaction theory
CMD: Common mental disorder
CMHP: Community Mental Health Program
DE: Design effect
LMIC: Low and Middle-Income Country
MOLISA: Ministry of Labour, Invalids and Social Affairs
OPC: Outpatient clinic
PA: Physician’s assistant
PHC: Primary Healthcare
PHP: Primary Healthcare Provider

## Footnotes

1 CHSs that were included in the interviews were excluded from the sampling frame.

2 Data available upon request

3 Many PHPs described the use of “tonics” or vitamins to improve blood circulation to the brain. This was noted in the context of reducing the somatic symptoms of mental illness, including headache.
