# Supplementary material for: Barriers and facilitators to the integration of depression services in primary care in Vietnam: a mixed methods study
Source: BMC Health Serv Res. 2018 Aug 16;18:641. doi: 10.1186/s12913-018-3416-z (PMC6097413; doi:10.1186/s12913-018-3416-z)
Supplement: Supplementary file 1 — Qualitative interview guide, this file contains the English language version of the semi-structured interview guide used to conduct the qualitative component of this study in Hanoi, Vietnam. (DOCX 17 kb) [file 12913_2018_3416_MOESM1_ESM.docx]

**Interview #____________________**

**Barriers and Facilitators to the Integration of Mental Health Services in Primary Care in Vietnam from the Perspective of Health Workers: Individual, Organizational and Structural Factors**

**Date: ________________________________**

**Sex of respondent: _________________________________**

**Introduction (to be read by interviewer):**

Thank you for agreeing to participate in this interview and for completing a consent form for the study entitled “Barriers and Facilitators to the Integration of Mental Health Services in Primary Care in Vietnam from the Perspective of Health Workers: Individual, Organizational and Structural Factors”. I’d like to remind you that I’m conducting this research as part of my PhD in Health Sciences at Simon Fraser University in Canada, and that I’m working in partnership with the Institute of Population, Health and Development in Hanoi.

My purpose with these interviews is to understand what factors might make it harder or easier for doctors and nurses working in commune health centres to integrate services for common mental disorders like depression into their everyday practice. With the help of my interpreter I’m going to ask you 16 questions about patients with mental health problems, your experience treating people with common mental disorders, about how you think about people with common mental disorders and treating them, about your training and about your work environment. The purpose of this study is not to assess the quality of primary care in Hanoi, but rather to understand the work and day to day experience of health workers. The overall goal of the study is to learn how to ensure that any steps to integrate mental health services into primary care are appropriate and meet the needs of health workers.

I’m going to start by asking you some general questions and will then ask you some more specific questions about working with patients with mental health problems, about your training and experience, and about your work load. I’d be happy to answer any questions you have before we begin.

What is your date of birth? __________________________

What is your professional role? ______________________________

How long have you worked in your current job? _________________________

Can you tell me a bit about a typical day for you? [Probing questions: When does it start? When does it end? Do you take breaks? How many patients do you see on an average day? What are the common health issues that you deal with? How long to do usually spend with a patient? Is it usually enough time? Do adult patients usually come alone or with family members?

**Part 1:**

1. Of the patients you see in an average month, how many would you say suffer from mental illness? What types of mental illnesses do they suffer from?
2. How significant an issue are common mental disorders like depression among patients visiting commune health centres?
3. What types of specific services, interventions or resources do people with common mental disorders like depression who visit commune health centres need?
4. What types of skills do people that treat people with mental disorders need to have to offer these services? What type of resources do they need to have?
5. Are there any challenges related to providing mental health services at this CHC? For example, do you have enough medications? What about training? What about working with the patients? Anything else?
6. In general, who is the most appropriate person to diagnose and treat people with depression?
7. Do you think that diagnosing and treating someone with depression is something that you can do? Do you think that diagnosing and treating people with depression should be part of your job? (Probing questions: do you think it is part of your role to work with patients with mental illness? Why? Why not? If not, what types of services and providers are more appropriate or more prepared?)
8. How do you think people in your community feel about people with common mental disorders like depression or anxiety? (Follow-up questions: what would they think causes depression or anxiety? What would they think about the character of someone with depression or anxiety? What type of opinion would they have about someone with depression or anxiety?)
9. What do people in your community think should be done about people who have mental health problems?
10. What do you think people in the health sector (like other people with the same job as you) community think about mental health? For example, what would they think about someone who decides to study psychiatry/ mental health nursing?

**Part 2:**

1. Tell me about the type of mental health training you received in medical/ nursing school. (Follow-up; What types of training? How much? Did you receive training on common mental disorders like depression and anxiety? Was it offered to everyone? If not, why did you choose to take it?)
2. Have you ever received professional development training on mental health? (If yes, can you please describe it? Why did you take it? Was it mandatory? Did your colleagues also take it?)
3. Does your commune health centre do anything specific to make mental health services available to patients? If yes, have they done this since you’ve been working here? If not, when did they start? What motivated them to start?
4. In general, what types of health issues would you say are given the most time and resources at this CHC? Why do you think these priorities have been chosen?
5. **OPTIONAL:** What types of health issues are prioritized on a national level? (Follow up: why do you think these issues have been prioritized? By whom? How do these priorities get identified? How do they play out in the day-to-day operations of a commune health centre (e.g. funding, training opportunities, available medicines?)
6. If you were offered the opportunity to strengthen your ability to diagnose and treat common mental disorders (e.g. additional training courses, use of new screening tools, for example) what would be your reaction? (Probing questions: do you feel like you have enough time to do this? Do you feel like these skills could fit in with the rest of your workload?) What types of information, support or resources would help?
