# Supplementary material for: Barriers and facilitators to the integration of depression services in primary care in Vietnam: a mixed methods study
Source: BMC Health Serv Res. 2018 Aug 16;18:641. doi: 10.1186/s12913-018-3416-z (PMC6097413; doi:10.1186/s12913-018-3416-z)
Supplement: Supplementary file 2 — Study survey instrument. (PDF 801 kb) [file 12913_2018_3416_MOESM2_ESM.pdf]

## Questionnaire

### "Barriers and Facilitators to the Integration of Mental Health Services in Primary Care in Vietnam from the Perspective of Health Workers: Individual, Organizational and Structural Factors".

Doctoral Research Study for PhD in Health Sciences, Faculty of Health Sciences, Simon Fraser University, Vancouver, B.C., Canada

Researcher: Gillian (Jill) Murphy, PhD Candidate, Simon Fraser University

***

Thank you for agreeing to complete this questionnaire. As described in the consent form, the purpose of this study is to understand the factors that may influence the success of programs to improve the integration of services for Common Mental Disorders such as depression and anxiety in commune health centres in Hanoi, Vietnam.

**While we use the term "Severe Mental Disorders" to refer to conditions such as schizophrenia and epilepsy, the term 'Common Mental Disorders' refers to conditions such as depression, anxiety and medically unexplained somatic symptoms (known as 'somatization') which are common in primary health care populations in many parts of the world.** . This questionnaire will take approximately 30 minutes to complete.

Please remember that your participation in this questionnaire is *completely voluntary*.

If you have any questions or concerns about the study you can contact me at: jgmurphy@sfu.ca or by phone at [-].

| | |
|---|---|
| **By filling out this questionnaire, you are consenting to participate: CONTINUE SURVEY**<br><br>**By filling out this questionnaire, you are consenting to participate: EXIT SURVEY** | |
| **Questions** | **Responses** |
| **We are happy to provide you with a small gift of 50,000 VND in phone credit for your participation in this survey. If you wish to receive this gift, please enter your cell phone number and the name of your cell phone provider (e.g. Viettel, Mobiphone, Vinaphone, etc.) We will send you the credit by SMS two or three days after completion of the survey.** | **Cell phone number:**<br><br>**Cell phone company name:** |

| | |
|---|---|
| 1: What is your professional role? | 1. Doctor<br><br>2. Doctor's Assistant<br><br>3. Other (please specify) |
| 2. What is your gender? | 1. Female<br><br>2. Male |
| 3. Please select the type of professional qualification you have. | 1. 6 year Medical degree<br><br>2. Masters Degree in Medicine (Please Specify Specialty)<br><br>3. Doctor's assistant college<br><br>4. Other (Please specify) |
| 4. In what year did you complete these qualifications? | Select from drop down menu |
| 5. During your training, was any time given to learning about mental disorders? | 1. Yes<br><br>2. No |
| 6. If you selected "yes" for #5, approximately **what percentage of time** was dedicated to learning about mental disorders during your training? | Fill in: |
| 7. Have you taken **additional training** about mental health as part of your professional development? | 1. Yes<br><br>2. No |
| 8. If you answered "yes" to #7, please indicate **when** (in years) you took this additional training. | 1. Within the last year<br><br>2. [Fill in] years ago |

| | |
|---|---|
| 9. If you answered "yes" to #7, please indicate **where** the training was offered. | 1. My commune health centre<br><br>2. My district health centre<br><br>2. Bach Mai Hospital/ National Institute of Mental Health<br><br>3. National Psychiatric Hospital #1<br><br>4.Other (please specify) |
| 10. Please select your level of agreement with the following statement:<br><br>In my current professional role, I would benefit from **more training** about mental disorders. | 1. Strongly agree<br><br>2. Somewhat agree<br><br>3. Somewhat disagree<br><br>4. Strongly disagree<br><br>5. Not applicable |
| 11: Approximately **how many patients** do you see during an average month? | Fill in: |
| 12. Of the patients you see in an average month, do you usually see any patients that suffer from a **mental illness**? | 1. Yes<br><br>2. No |
| 13. If you selected "yes" for #12, **approximately how many** patients do you see during an average month that suffer from a mental illness? | Fill in: |
| 14. If you selected "yes" for #12, approximately how many patients do you see during an average month whose **primary reason for consultation** is a mental illness? | Fill in: |

| | |
|---|---|
| 15. If you selected "yes" for #12, approximately how many of the patients that you see in an average month suffer specifically from a **Severe Mental Disorder** (e.g. schizophrenia, epilepsy)? | Fill in: |
| 16. If you selected "yes" for #12, approximately how many of the patients that you see in an average month suffer specifically from a **Common Mental Disorder** (e.g. depression anxiety or and somatization)? | Fill in: |
| | |
| 17. In your opinion, how widespread are Common Mental Disorders like depression among the patients that visit your Commune Health Centre? | 1. Very widespread<br>2. Moderately widespread<br>3. Not widespread<br>4. Not applicable |
| 18. In your opinion, how widespread are Common Mental Disorders like depression in the community in general? | 1. High prevalence<br>2. Moderately high prevalence<br>3. Moderately low prevalence<br>4. Very low prevalence<br>5. Not applicable |
| 19. How would you rate the severity of Common Mental Disorders like depression in terms of the impact they have on the lives of patients or people in the community (e.g. ability to perform work, daily tasks and maintain important relationships)? | 1. High severity<br>2. Moderate severity<br>3. Moderately low severity |

| | |
|---|---|
| | 4. Low severity<br><br>5. Not applicable |
| 20. Please select your level of agreement with the following statement:<br><br>Commune health centres are the **appropriate** place for people with depression and other Common Mental Disorders to receive care. | 1. Strongly agree<br><br>2. Somewhat agree<br><br>4. Somewhat disagree<br><br>5. Strongly disagree |
| 21. Reflecting on your own experience, how **confident** are you that you can **diagnose** a patient with a Common Mental Disorder such as depression? | 1. Very confident<br><br>2. Somewhat confident<br><br>3. Not very confident<br><br>4. Not at all confident<br><br>5. Not applicable |
| 22. Reflecting on your own experience, how **confident** are you can **effectively treat** a patient with a Common Mental Disorder such as depression? | 1. Very confident<br><br>2. Somewhat confident<br><br>3. Not very confident<br><br>4. Not at all confident<br><br>5. Not applicable |
| 23. Please select your level of agreement with the following scenario:<br><br>Your friend just told you that he suffers from depression.<br><br>You want to spend time with him socially and continue to invite him to social events. | 1. Strongly agree<br><br>2. Somewhat agree<br><br>3. Somewhat |

| | |
|---|---|
| | disagree<br><br>4. Strongly disagree |
| 24. Please select your level of agreement with the following scenario:<br><br>Your sister-in-law complains of headaches and pain in her limbs that could not be diagnosed medically and which has affected her ability to perform daily tasks.<br><br>You wonder if she is just being lazy. | 1. Strongly agree<br><br>2. Somewhat agree<br><br>3. Somewhat disagree<br><br>4. Strongly disagree |
| 25. Please select your level of agreement with the following scenario:<br><br>Your neighbour is fearful of leaving his house and has been diagnosed with an anxiety disorder.<br><br>You feel uncomfortable with him living nearby. | 1. Strongly agree<br><br>2. Somewhat agree<br><br>3. Somewhat disagree<br><br>4. Strongly disagree |
| 26. Please select your level of agreement with the following scenario:<br><br>Your colleague has recently returned to work after taking time off to undergo treatment for depression.<br><br>You are confident in her ability to do her job effectively. | 1. Strongly agree<br><br>2. Somewhat agree<br><br>3. Somewhat disagree<br><br>4. Strongly disagree |
| 27. Does your commune health centre have **a specific protocol for** providing mental health services to patients (e.g. diagnosis, training)? | 1. Yes<br><br>2. No<br><br>4. Don't know |
| 28. Does your commune health centre have a **specific protocol** for providing referrals to patients with mental health problems? | 1. Yes |

| | |
|---|---|
| | 2. No<br><br>4. Don't know |
| 29. Have you been involved in discussion(s) with other coworkers about providing mental health services to patients? | 1. Yes<br><br>2. No |
| 30. If you answered "yes" to #29 , did the discussion(s) take place in a formal meeting? | 1. Yes<br><br>2. No |
| 31 . If you answered "yes" to #29, did the discussion(s) take place informally (e.g. among colleagues at lunch or during a break)? | 1. Yes<br><br>2. No |
| 32 . If you answered "yes" to #29, did the discussion(s) include managers or directors of the commune health centre? | 1. Yes<br><br>2. No |
| 33. If you identified that a patient was suffering from a Common Mental Disorder such as depression, what would you do? | 1. Refer them to the district health centre<br><br>2. Refer them to a general hospital<br><br>3. Refer them to a psychiatric hospital<br><br>4. Prescribe medication<br><br>5. Other (please specify) |
| 34. When patients are prescribed medications for Common Mental Disorders such as depression, where would they usually access these medications? (Chose all that apply) | 1. From a pharmacist at the commune health centre<br><br>2. From a private pharmacy<br><br>3. From a hospital |

| | |
|---|---|
| | pharmacy<br><br>4. Other (please specify) |
| 35. When patients are prescribed medications for Common Mental Disorders such as depression (e.g. antidepressant medications), are you concerned about their ability to access these medications? | 1. Yes<br><br>2. No<br><br>3. Sometimes |
| 36 . If you answered "yes" or "sometimes" to #35, why wouldn't patients **be able to access** these medications? (Chose all that apply) | 1. They are unavailable in the Commune Health Centre pharmacy<br><br>2. They are unavailable at private pharmacies.<br><br>3. They are too expensive for patients to afford<br><br>3. Other (please specify) |
| 37 . Is **privacy** (e.g. an examination room where no other patients can see or overhear) necessary for consulting with patients with Common Mental Disorders such as depression? | 1. Yes<br><br>2. No<br><br>3. Sometimes<br><br>4. Not applicable |
| 38 . If you answered "yes" or "sometimes" to #37, does your commune health centre have **private space available** to allow for effectively consulting with patients with Common Mental Disorders such as depression? | 1. Yes<br><br>2. No<br><br>3. Sometimes |
| 39 . Please select your level of agreement with the following statement:<br><br>Our commune health centre would be able to provide better care to people with Common Mental Disorders such as depression if **we had more staff.** | 1. Strongly agree<br><br>2. Somewhat agree<br><br>3. Somewhat |

| | |
|---|---|
| | disagree<br><br>4. Strongly disagree<br><br>5. Not applicable |
| 40 . Please select the most accurate response to the following statement:<br><br>The **number of patients** I see each day makes my workload difficult to manage. | 1. Always<br><br>2. Sometimes<br><br>3. Never<br><br>4. Not applicable |
| 41 . Please select the most accurate response to the following statement:<br><br>The **number of patients** I see each day causes me stress. | 1. Always<br><br>2. Sometimes<br><br>3. Never<br><br>4. Not applicable |
| 42 . Please select the most accurate response to the following statement:<br><br>I wish I were able to spend more **time** during consultations with my patients. | 1. Always<br><br>2. Sometimes<br><br>3. Never<br><br>4. Not applicable |
| 43 . Please select your level of agreement with the following statement:<br><br>The amount of **paper work** I have each day makes my workload difficult to manage. | 1. Always<br><br>2. Sometimes<br><br>3. Never<br><br>4. Not applicable |
| 44. Please select your level of agreement with the following statement: | 1. Always |

| | |
|---|---|
| My responsibilities related to **specific programs** makes my workload difficult to manage. | 2. Sometimes<br><br>3. Never<br><br>4. Not applicable |
| 45. Please select your level of agreement with the following statement:<br><br>The amount of **paper work** I have each day causes me stress. | 1. Always<br><br>2. Sometimes<br><br>3. Never<br><br>4. Not applicable |
| 46. Please select your level of agreement with the following statement:<br><br>Given my current workload, I feel confident that I could take the time to **learn new skills.** | 1. Strongly agree<br><br>2. Somewhat agree<br><br>3. Somewhat disagree<br><br>4. Strongly disagree<br><br>5. Not applicable |
| 47 . Please select your level of agreement with the following statement:<br><br>Given my current workload, I feel confident that I could **put new skills into practice.** | 1. Strongly agree<br><br>2. Somewhat agree<br><br>3. Somewhat disagree<br><br>4. Strongly disagree<br><br>5. Not applicable |
| 48. Please select your level of agreement with the following statement:<br><br>I would be reluctant to learn and implement new skills without being **offered an increase in my salary or benefits.** | 1. Strongly agree<br><br>2. Somewhat agree<br><br>3. Somewhat |

| | |
|---|---|
| | disagree<br><br>4. Strongly disagree<br><br>5. Not applicable |
| 49. Please select your level of agreement with the following statement:<br><br>I would **like to learn** more about diagnosing and treating people with Common Mental Disorders such as depression. | 1. Strongly agree<br><br>2. Somewhat agree<br><br>3. Somewhat disagree<br><br>4. Strongly disagree |
| 50. Is there is a person at your commune health centre who has taken a **leadership role** to improve services for people with Common Mental Disorders such as depression? | 1. Yes<br><br>2. No<br><br>3. Don't know |
| 51. As far as you know, is there a national policy about mental health? | 1. Yes<br><br>2. No<br><br>3. Don't know |
| Please feel free to provide any additional comments that you think might be relevant to this study: | |
